# Estimating the window of selection of antimalarial drugs using field data

**DOI:** 10.1186/1475-2875-11-S1-O33

**Published:** 2012-10-15

**Authors:** Katherine Winter, Ian Hastings

**Affiliations:** 1 Liverpool School of Tropical Medicine, Liverpool, L3 5QA, UK

## 

Drug resistance spreads through a population because (i) parasites can survive direct treatment and/or (ii) survive “residual” drug levels persisting from previous treatment. High levels of drug use and long drug half-lives mean a high proportion of the population may have residual drug levels selecting for resistance. Field studies can quantify this second force of resistance by estimating a window of selection (WoS); a period of time during which drug levels have declined sufficiently to allow new infections with drug resistant parasites while still able to prevent infections by drug sensitive parasites. Typically this is estimated by comparing how early different genotypes become detectable after treatment. These WoS estimates are widely cited and we refer to them as ‘apparent’ WoS to denote their origin in clinical observations. In fact the ‘true’ WoS is the period in which infections bearing the mutation can emerge from the liver and survive to produce a patent infection while sensitive parasites are killed by residual drug concentrations. Unfortunately, it is impossible to observe this emergence because parasites (assuming 105 emerge) are below the microscopic limit of detection (assumed to be 10^8^) and so we are forced to rely on ‘apparent’ WoS i.e., the time at which they have grown to patency.

We use the validated mechanistic pharmacokinetic-pharmacodynamic (PK/PD) model described in Winter & Hastings [1] to simulate WoS for increasingly resistant infections and to ascertain how accurately field observations estimate the ‘true’ window of selection. Observed estimates of the WoS generally provide a good match to the ‘true’ WoS when resistance levels are moderate to high (i.e., increases in the half-maximal killing rate or IC50 of 10-fold or more). However, field methods may overestimate it when resistance levels are low or when parasites are able to successfully cause reinfections in the first few days following treatment. The latter is usually accompanied by high levels of resistance and hence treatment failures that should provide warnings that this effect may occur.
